# Cognitive pathways to the forms and functions of aggression in adolescence: the role of early maladaptive schemas and social information processing

**DOI:** 10.3389/fpsyg.2025.1431756

**Published:** 2025-03-13

**Authors:** Paula Vagos, Matteo Angelo Fabris, Daniel Rijo

**Affiliations:** ^1^Department of Education and Psychology, William James Center for Research, University of Aveiro, Aveiro, Portugal; ^2^Faculty of Psychology and Educational Sciences, Center for Research in Neuropsychology and Cognitive Behavioral Intervention, University of Coimbra, Coimbra, Portugal; ^3^Department of Psychology, University of Turin, Turin, Italy

**Keywords:** early maladaptive schemas, social information processing, hostile attribution of intent, response evaluation, forms and functions of aggression

## Abstract

**Introduction:**

Early maladaptive schemas (EMSs) have been found to associate to aggressive behavior, though the cognitive pathways underlying that association remain scarcely investigated, particularly considering the different forms and functions of aggression. The current work explores the sequential mediation pathways linking EMSs and variables related to social information processing (SIP; i.e., hostile attribution of intent and evaluation of overt and relational responses) to aggressive behaviors.

**Methods:**

A sample of 516 adolescents (M_*age*_ = 16.54, 69.4% female) filled in self-report questionnaires on EMSs, SIP, and the forms and functions of aggression. A model generation approach based on retaining only significant direct pathways was applied to four mediation models that differed in the outcome variable: reactive overt aggression, proactive overt aggression, reactive relational aggression, and proactive relational aggression.

**Results:**

Results showed the salience of EMSs within the disconnection and rejection and the impaired limits domains and of a positive evaluation of aggressive response options. Alternatively, specific results were found for hostile attribution of intent in relation to relational aggression regardless of its function, for reactive overt aggression, and for proactive overt aggression (e.g., hostile attribution of intention impacted indirectly on relational aggression, directly on reactive overt aggression and did not impact on proactive overt aggression).

**Discussion:**

Overall and specific findings are discussed based on both developmental (e.g., early neglectful or punitive experiences) and current interaction processes (e.g., social or personal gains associated with the practice of aggressive behavior). Overall, adolescent aggression seems sustained by cognitive pathways that may be more malleable to change based on joint intra and interpersonal intervention efforts.

## 1 Introduction

Cognitive schemas have been defined as cognitive themes, patterns or assumptions that individuals hold true about themselves, others, and interactions and that are used to make sense of ones’ life experiences. Though schemas may be thought of as flexible and adaptable in order to accurately represent ones’ contextual experiences (Steffen et al., 2017), within the Schema Therapy framework schemas are proposed as pervasive maladaptive patterns that tend to be extreme, biased and often negative representations that, when triggered by a given situation, elicit schema-congruent emotional and behavioral responses. These Early Maladaptive Schemas (EMSs) are developed early in life (i.e., they were once an accurate representation of ones’ experiences) and often remain stable because, in any situation relevant for a specific EMS, schema related processes influence information processing in a way that prompts individuals to retain evidence favoring the EMS and to neglect information that may contradict it (Rafaeli et al., 2010). Though Young et al. (2006) proposed that EMSs develop during childhood or adolescence and are continuously elaborated throughout ones’ life, evidence has shown that EMSs are applicable to adolescents (Van Vlierberghe et al., 2010), namely within the framework of 18 EMSs grouped into five schema domains (e.g., Borges et al., 2020; Santos et al., 2018); for a description of the EMSs and their respective domains see Supplementary Table A. Moreover, EMSs have been consistently associated with measures of psychopathology in adolescents, namely depressive and anxious symptoms, eating disorders borderline personality symptoms, and externalizing behaviors (Nicol et al., 2020), including the practice of aggressive behavior. Still, the cognitive pathways that link EMS to aggressive behavior have seldom been explored.

Such pathways may be conceptualized within the Social Information Processing (SIP; Crick and Dodge, 1994) model, which has been applied to understanding the practice of social behaviors, including aggressive behaviors. Though not explicitly referring to EMSs, the SIP model proposes that schemas sustain the way information taken from social situations is initially encoded and represented (i.e., what meaning is attributed to the available information). This meaning, when based on biased or inflexible negative schemas, may also be biased wherein the individual assumes that others have an hostile intent towards them, including to provoke, hurt, humiliate or in any other way cause damage to the individual (i.e., hostile attribution of intent). The SIP model proposes that this meaning is subsequently recalled in future interactions and influences the way individuals evaluate different behavioral options and come to choose and enact one of those options that both follows from the intention they attribute to others and that more closely allows them to attain specific interaction goals, including instrumental (e.g., money) or social gains (e.g., prestige). In other words, a positive evaluation of a behavior substantiates the acting of that behavior (Crick and Dodge, 1994). The premises of the SIP model make it plausible to assume cognitive pathways linking EMS to a biased (hostile) attribution of meaning to an event, in light of which the individual would favor and ultimately enact an aggressive response.

Previous evidence has shown the association of each of these aspects with aggressive behavior. About the association between core cognitive schemas and aggressive behavior in adolescence, previous findings sustain that grandiosity longitudinally predicts the practice of aggressive behavior (Calvete, 2008). EMSs within the disconnection and rejection domain have been proposed as ways though which exposure to family violence may lead to dating violence in community adolescent samples (Calvete et al., 2018b) and to externalizing behavior within a sample of adolescents diagnosed with oppositional defiant, conduct, or disruptive behavior disorder not otherwise specified (Schilder et al., 2021). Also, adolescents who endorsed higher levels of core cognitive themes about obedience, emotional inhibition and strict standards also referred to practicing verbal and non-verbal aggressive behavior (Vatani and Namdarpour, 2022). None of these works considered the framework of 18 EMSs proposed by Young et al. (2006). About the attribution of meaning, aggressive behavior has been consistently associated with a hostile attribution of intent to others’ actions, particularly in more aggressive individuals and when information is collected based on real social interactions (de Castro et al., 2002). As for response evaluation of aggressive behaviors, adolescents who find violence justifiable (Calvete, 2008) or who hold positive evaluations of aggressive responses to ambiguous provocations (Fontaine et al., 2009) also tend to report more antisocial or aggressive behavior.

Though these are relevant findings, they still lack the consideration of a sequential pathway linking EMSs, attribution of intent, and response evaluation to the practice of aggressive behavior. Other works have considered this sequence in adolescent samples, particularly using longitudinal designs. Fontaine et al. (2010) considered adolescents transitioning from grade 11 to grade 12 and focused on the link between hostile attribution bias, response evaluation and antisocial behavior. Their findings showed that a positive evaluation aggression fully mediated the association between hostile attribution of intent and antisocial behavior practiced roughly one year later. Still, these authors did not consider EMSs. In turn, Calvete and Orue (2012) found that core cognitive schemas referring to mistrust, narcissism and justification of violence had differential effects on SIP-related variables roughly 6 months later: mistrust predicted a hostile interpretation of ambiguous situations and feelings of anger; narcissism explained feelings of anger and recalling an aggressive response option; and justification of violence only impacted on recalling that response option. Finally, accessing an aggressive response option impacted on reacting aggressively about 1 year later. Still, core cognitive themes identified by Calvete and Orue (2012) do not necessarily align with the EMSs framework. Finally, neither of these works considered aggressive behavior based on its diverse forms and functions, though such conceptualization has been found to apply to adolescents within varying contexts (Marsee et al., 2014; Polman et al., 2007).

Aggressive behavior may be conceptualized based on two forms (i.e., overt versus relational) and two functions (i.e., reactive versus proactive), which, in turn, may combine into four ways of acting aggressively: reactive overt aggression, proactive overt aggression, reactive relational aggression, and proactive relational aggression. About the forms of aggression (i.e., the “what” of aggression), overt aggression refers to aggressive acts intending to directly cause damage to the victim or to their possessions, whereas relational aggression causes damage to the victim indirectly by damaging their relationship with others or their social status within the group. As for the functions of aggression (i.e., the “why” of aggression), reactive aggression is elicited as a reaction to a real or perceived threat or provocation from others, while proactive aggression occurs as a chosen act that the individual believes is the best way to achieve their interaction goals (Fiske et al., 2010; Little et al., 2003). The functions of aggression in particular have been associated with mental health related outcomes, including social information processing related variables (Khouwaga Yusoufzai and Lobbestael, 2022). Previous review works concur on reactive (but not proactive) aggression being associated with a tendency to attribute hostile intention to others (Hubbard et al., 2010; Martinelli et al., 2018); alternatively, proactive (but not reactive) aggression was linked to a positive evaluation of aggression as an effective mean of achieving ones’ interpersonal goals (Hubbard et al., 2010), as well as with endorsing agentic/self-focused goals in interpersonal contexts, which in turn was based on holding positive views about the self (Salmivalli et al., 2005). About the forms of aggression, the association between relational or overt aggression and hostile attribution of intent seems to be particularly relevant when that attribution is made to coherent (i.e., relational or overt) ambiguous provocations (Martinelli et al., 2018), although evidence also exists of overall hostile attribution of intent associating with relational aggression in both its functions (Kokkinos et al., 2017). Moreover, rating aggression as an effective response in problematic social situations significantly accounts for the intention of acting aggressively, particularly when the form of aggression is coherent between evaluation and attribution of intention (Farrell and Bettencourt, 2020). None of these works considered the relevance of EMSs as the starting point of cognitive pathways leading to diverse ways of acting aggressively.

The current work intends to address those cognitive pathways, by exploring the sequential effect of EMSs, hostile attribution of intent and evaluation of aggressive response (these last two considered as SIP-related variables) on the practice of aggressive behavior. It adds to previous works in that it considers the 18-EMSs conceptual framework and the four possible combinations of aggressive behavior as outcomes. Specifically, four mediation models were analyzed wherein the direct and indirect effects of EMSs and SIP-related variables were investigated in relation to reactive overt aggression (i.e., Model 1), proactive overt aggression (i.e., Model 2), reactive relational aggression (Model 3), and proactive relational aggression (i.e., Model 4). Previous literature has shown that the association between SIP-related variables and aggression may diverge based on the functions (e.g., Hubbard et al., 2010) and forms (e.g., Martinelli et al., 2018) of aggression and no previous works considered EMSs in relation to SIP-related variables and to the forms and functions of aggression. So, specific models may make noticeable cognitive specificities applicable to each combination of the forms and functions of aggression as outcomes. Based on previous findings, we expect that EMSs related to the disconnection and rejection (Calvete et al., 2018b) and to the impaired limits (i.e., grandiosity; Calvete, 2008) domains, as well as EMSs about obedience (i.e., subjugation) and emotional inhibition (Vatani and Namdarpour, 2022) may be particularly salient and directly/ indirectly impact aggressive behavior, particularly overt aggression, which was the form addressed in previous works. Also, we expect hostile attribution of intent to be particularly associated with reactive (overt and relational) aggression (Martinelli et al., 2018), and that a positive evaluation of a relational/ overt aggressive response will relate to practicing that response (Farrell and Bettencourt, 2020). Finally, we expect that SIP-related variables will independently and sequentially mediate the association between EMSs and aggressive behavior: the more individuals attribute hostile intentions to others and/or positively evaluate aggressive behavior, the more they report practicing that behavior (Calvete and Orue, 2012).

## 2 Materials and methods

The study involved humans and was approved by the Institutional Review Board of Direção Geral da Educação – Ministério da Educação (Inquiry number 0170100008). The study was conducted in accordance with the local legislation and institutional requirements. Written informed consent for participation in this study was provided by the participants’ legal guardians/next of kin.

### 2.1 Participants

Seven schools located in the north and center regions of Portugal were contact based on their position in the national ranking of schools, which is based on students’ academic achievement: two schools presented below average results, three schools offered within average results and two schools obtained above average results. Schools were asked to collaborate in this research by serving as intermediates between the research team and both adolescents and their parents/legal guardians. Written informed consent forms were sent to parents/legal guardians after adolescents themselves were informed on the goals and procedures of this research (i.e., to understand aggressive behavior in adolescents, including its association with psychological processes and with other social behaviors). Adolescents were also informed on the confidentiality and anonymity of their data, and of the voluntary nature of their participation; no further incentives were given. Inclusion criteria were as follows: (1) adolescents attending the 10*^th^* through 12*^th^* grades and (2) adolescents whose parents/legal guardians gave written informed consent and who themselves consented to their own participation. Exclusion criteria included adolescents who were signaled for specific education needs that could impact on the understanding and filling in of the self-report instrument used for data collection. Data collection took place in the classroom in time made available by the teacher; the teacher and one member of the research team were available to assist and clarify doubts if needed.

The final sample consisted of 516 adolescents aged 15–19 years old (M == 16.54, SD = 1.15), of which 69.4% (*n* = 358) were female and 30.6% (*n* = 158) were male. They attended the 10*^th^* (*n* = 219, 42.4%), 11*^th^* (*n* = 130, 25.2%) or 12*^th^* grades (*n* = 167, 32.4%). Most participants had never been retained in the same school year before (*n* = 357, 69.2%), and came from an intact family [i.e., living with parent(s) and siblings if having any; *n* = 488, 94.6%] who belonged to a low socioeconomic status (*n* = 272, 52.7%). Female and male participants had similar mean ages [*t*_(514)_ = −1.06, *p* = 0.29]. Also, female and male participants were evenly distributed based on their history of previous school holdbacks [χ^2^_(1)_ = 0.19, *p* = 0.66] and family’s socioeconomic status [χ^2^_(2)_ = 1.68, *p* = 0.43]. Alternatively, female and male participants were not distributed similarly across schools grades [χ^2^_(2)_ = 9.28, *p* = 0.01], with males being less prevalent then statistically expected in the 11*^th^* and 12*^th^* grades.

### 2.2 Instruments

All instruments were used in their Portuguese version.

#### 2.2.1 Young schema questionnaire for adolescents—brief form (B-YSQ-A)

The B-YSQ-A proposed by Santos et al. (2018) consists of 54 items selected based on statistical (i.e., inter-item and item-total correlations) and theoretical premises (i.e., expert rating of closeness of content) out of the 90 items composing the adult version of the Young Schema Questionnaire (Young, 2005). Those 54 items were selected to address the 18 EMS (i.e., three items addressing each EMS) and are rated using a six-point scale ranging from 1 (has nothing to do with me) to 6 (it is exactly what happens to me); for examples of items see Supplementary Table A. Previous works used confirmatory factor analyses to ascertain the internal structure of the instrument as organized into 18-correlated dimensions, which was invariant across male and female adolescents (Santos et al., 2018) and across Portuguese and Brazilian participants (Borges et al., 2020). The 18 dimensions corresponding to 18 EMSs had at least acceptable internal consistency values (i.e., α between 0.63 and 0.88; Borges et al., 2020; Santos et al., 2018), test-retest reliability (*r*^2^ between 0.44 and 0.77), and construct validity in relation to internalizing and externalizing symptoms (Santos et al., 2018) and in relation to anxiety, depression, and stress symptoms and well-being (Borges et al., 2020). Considering the current sample, most dimensions within the B-YSQ-A achieved at least acceptable internal consistency values, ranging from α = 0.61 for mistrust/abuse to α = 0.87 for failure. Alternatively, measures for unrelenting standards (α = 0.57) and grandiosity (α = 0.54) achieved questionable internal consistency values. Nevertheless, because (1) the Cronbach alpha is dependent on the number of items (i.e., scales with less items are expected to present lower values; Streiner, 2003), (2) this study focuses on an explanatory theoretical model (vs applied to clinical issues), and (3) some of these schemas may be particularly relevant to aggressive behavior (e.g., grandiosity; Calvete, 2008), we chose not to exclude these subscales from the analyses.

#### 2.2.2 Scenes for social information processing for adolescents (SSIPA)

The SSIPA (Vagos et al., 2016) includes six hypothetical scenarios representing ambiguous relational and overt interactions. The individual is asked about several aspects in relation to those situations, as to address diverse steps of the social information processing model (Crick and Dodge, 1994), including attribution of intent, emotional arousal, evaluation of diverse behaviors based on several criteria proposed by Fontaine and Dodge (2006), and likelihood of practicing those same behaviors. Exploratory factor analyses revealed two measures for attribution of intent (i.e., hostile and neutral), three measures for emotional arousal (i.e., anger, shame and sadness), positive evaluation of passiveness, assertiveness, overt aggression and relational aggression in situations of overt and relational provocation, and endorsement of the practice of passiveness, assertiveness, overt aggression and relational aggression. For the purpose of the current work, which focused on assessing the cognitive pathways underlying aggressive behavior in its various forms and functions, we used only the measures pertaining to hostile attribution of intent and the positive evaluation of overt and relational aggression. The hostile attribution of intent measure consists of five items (e.g., ‘People don’t like me and don’t want me on their team’) and the respondent is asked to rate their likelihood from 1 (“not at all likely”) to 5 (“very likely”). This subscale was defined via exploratory factor analyses, attained a good internal consistency value (α = 0.72), and was further confirmed as applicable invariantly to male and female adolescents. The positive evaluation of relational and overt aggression measures include 12 items each and were derived from both theoretical assumptions and exploratory factor analyses outcomes. Each measure considers several evaluation criteria (e.g., self-efficacy in practicing that response addressed by asking “How capable are you of…”) concerning relational (e.g., “When no one is watching, complain to my coach or teacher”) or overt (e.g., “Tell them: You’d better pick me next time or else…”) aggression applicable to scenes of overt and relational provocation. Evaluation measures were confirmed as invariantly applicable to male and female adolescents and achieved very good internal consistency values (α ≥ 0.92). SSIPA measures used in this work also achieved at least good internal consistency values using the current sample: α = 0.79 for hostile attribution of intent, α = 0.94 for positive evaluation of overt aggression, and α = 0.95 for positive evaluation of relational aggression.

#### 2.2.3 Peer conflict scale [PCS]

The PCS includes 40 items describing the practice of aggressive acts under different forms and functions, to which the adolescent responds using a one (not at all true) to four (definitely true) scale in its Portuguese Version (Vagos et al., 2014). Previous evidence based on confirmatory factor analyses has shown the internal structure of the PCS to be organized into four measures of aggressive behavior: reactive overt aggression (e.g., “When I am teased, I will hurt someone or break something”), proactive overt aggression (e.g., “I have hurt others to win a game or contest”), reactive relational aggression (e.g., “I gossip about others when I’m angry at them”), and proactive relational aggression (e.g., “I deliberately exclude other from my group, even if they haven’t done anything to me”; Marsee et al., 2011; Vagos et al., 2014). This measurement model was found to be invariant between male and female participants (Marsee et al., 2011; Vagos et al., 2014) and between community, detained and residential participants (Marsee et al., 2011). Moreover, those measures presented good internal consistency values (i.e., α between 0.70 and 0.91; Marsee et al., 2011; Vagos et al., 2014), and construct validity in relation to self-reported delinquency (Marsee et al., 2011). Using the current sample, all measures of aggression achieved at least good internal consistency values varying between α = 0.80 for reactive relational aggression and α = 0.89 for reactive overt aggression.

### 2.3 Data analyses

Preliminary analyses were conducted on the association between EMSs measures and the selected SIP-related mediation variables (i.e., hostile attribution of intent and evaluation of relational or overt aggression) using the IBM SPSS Statistics 26 software (IBM Corp, 2019); only EMSs that correlated significantly with one or both of SIP-related variables and/or with our aggresion were selected for the mediation analyses. Four sequential mediation models were tested using Mplus v7.0 (Muthén and Muthén, 2012), which differed in the aggressive behavior being considered as the outcome variables: reactive overt aggression (i.e., Model 1), proactive overt aggression (i.e., Model 2), reactive relational aggression (Model 3), and proactive relational aggression (i.e., Model 4). All models included EMSs as independent variables and hostile attribution of intent and evaluation of response as sequential mediators. For Models 1 and 2, evaluation of overt aggression was used and for Models 3 and 4 evaluation of relational aggression was entered. The general depiction of these models is presented in Supplementary Figure A. A model-generation approach was applied to each model: non-significant pathways were excluded one at a time based on having the highest non-significant *p*-values; whenever excluding pathways led to a variable not contributing to explaining the variance of our outcomes (either directly or indirectly), that variable was excluded from the model at that time. We opted to exclude one pathway at a time because each exclusion might have implications for the remaining pathways that would not be noted otherwise. Detailed description of excluded pathways and variables, in the order they were excluded, will be made available from the corresponding author, upon request and without undue reservation. No additional pathways were added throughout this model generation approach as they would collide with the theoretical assumptions we aimed to test (i.e., that EMSs contribute both directly and indirectly – via SIP-related variables – to aggression, and that SIP-related variables also sequentially impacted in aggression; Supplementary Figure A). This approach was applied to each model so that resulting models could be both statistically and theoretically useful, as well as comprehensive in relation to retention of relevant independent and mediator variables.

The fit of the model was assessed based on a two-index approach proposed by Hair et al. (2009). Specifically, we combined an acceptable value for the Comparative Fit Index value (i.e., CFI ≥ 0.95) with either an acceptable value for the Root Mean Square Error of Approximation (i.e., RMSEA ≤ 0.07) or an acceptable value for the Standardized Root Mean Square Residual (i.e., SRMR ≤ 0.08). The magnitude and direction of direct and indirect effects, as well as the variance explained of each of mediating variables and of the outcome variable, were also considered.

Missing data represented 1.54% of the possible data pool as was not missing completely at random (MCAR *χ*^2^_(1132)_ = 1276.06, *p* = 0.002). The Maximum Likelihood Robust estimator was used for dealing with the missing data and deviations from the multivariate normal distribution (Mardia’s Skewness *z* = 6690.88, *p* < 0.001 and Mardia’s Kurtosis *z* = 33.77, *p* < 0.001).

## 3 Results

### 3.1 Preliminary correlation analyses

Detailed results on these preliminary analyses are presented in Supplementary Table B. Most schemas (i.e., emotional deprivation, abandonment/instability, mistrust/abuse, social isolation/alienation, defectiveness, failure, dependence/incompetence, vulnerability to harm and illness, entitlement/grandiosity, insufficient self-control/discipline, subjugation, approval/recognition-seeking, emotional inhibition, unrelating standards/hypercriticalness, negativity/pessimism and punitiveness) correlated significantly and positively with hostile attribution of intent, with values ranging from *r*_*s*_ = 0.12, *p* = 0.006 for grandiosity to *r*_*s*_ = 0.42, *p* < 0.001 for defectiveness/shame.

Only eight EMSs correlated significantly and positively with positive evaluation of overt aggression (i.e., emotional deprivation, mistrust/abuse, social isolation/alienation, defectiveness/shame, grandiosity, insufficient self-control/discipline, approval/recognition seeking, and negativity/pessimism), with value ranging from *r*_*s*_ = 0.14, *p* = 0.004 for negativity/pessimism to *r*_*s*_ = 0.36 *p* < 0.001 for insufficient self-control/discipline; in turn positive evaluation of overt aggression correlated significantly but negatively with self-sacrifice (*r*_*s*_ = −0.11, *p* = 0.02). Otherwise, most EMS correlated significantly and positively with positive evaluation of relational aggression (i.e., emotional deprivation, abandonment/instability, social isolation/alienation, defectiveness/shame, dependence/incompetence, entitlement/ grandiosity, insufficient self-control/discipline, subjugation, self-sacrifice, and approval/recognition-seeking), with correlation values ranging from *r*_*s*_ = 0.13, *p* = 0.006 for subjugation to *r*_*s*_ = 0.31, *p* < 0.001 for insufficient self-control/discipline; in turn, abandonment/instability (*r*_*s*_ = −13, *p* = 0.005) and self-sacrifice (*r*_*s*_ = −18, *p* < 0.001) had significant but negative correlation values with positive evaluation of relational aggression.

Most EMSs correlated with at least one measure of aggressive behavior and all significant correlation values were positive. Specifically, higher reactive overt aggression and higher proactive overt aggression associated with higher emotional deprivation, mistrust/abuse, social isolation/alienation, defectiveness/shame, vulnerability to harm or illness, entitlement/grandiosity, insufficient self-control/discipline, approval/recognition-seeking, unrelating standards/hypercriticalness, and negativity/pessimism; higher proactive (but not reative) overt aggression also correlated significantly with subjugation. Correlation values ranged from *r*_*s*_ = 0.10, *p* = 0.03 for emotional deprivation to *r*_*s*_ = 0.29, *p* < 0.001 for insufficient self-control/discipline concerning reactive overt aggression and from *r*_*s*_ = 0.10, *p* = 0.03 for subjugation to *r*_*s*_ = 0.33, *p* < 0.001 for insufficient self-control/discipline for proactive overt aggression. Both reactive and proactive relational aggression correlated significantly with emotional deprivation, mistrust/abuse, social isolation/alienation, defectiveness/shame, failure, dependence/incompetence, vulnerability to harm or illness, entitlement/grandiosity, insufficient self-control/discipline, subjugation, approval/recognition-seeking, emotional inhibition, unrelating standards/hypercriticalness, and negativity/pessimism. Correlation values ranged *r*_*s*_ = 0.10, *p* = 0.03 for failure to *r*_*s*_ = 0.37, *p* < 0.001 for grandiosity in relation to reactive relational aggression and from *r*_*s*_ = 0.13, *p* = 0.005 for unrelenting standards/ hyper criticalness to *r*_*s*_ = 0.30, *p* < 0.001 for insufficient self-control/discipline regarding proactive relational aggression.

Based on these findings, only the enmeshment/undeveloped self was dropped from the mediation analyses, as it did not correlate with any of the mediators or the outcome variables.

### 3.2 Analyses of mediation models

**FIGURE 1:**
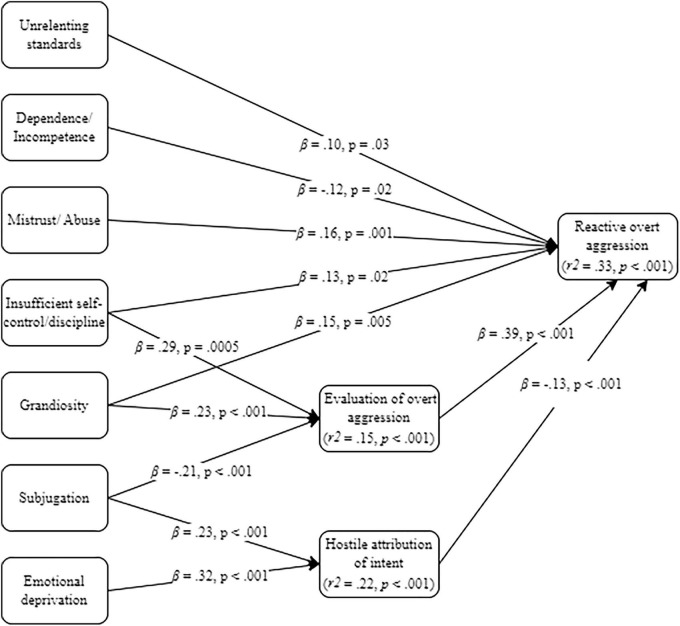
Generated mediation model on early maladaptive schemas (EMS), Social Information Processing (SIP)-related variables and reactive overt aggression.

All baseline models (see Supplementary Figure A) were just identified. The generated models always achieved acceptable fit indicators (see Table 1) and, aligned with our model-generation strategy, included only significant direct (see Figure 1 through 4) and indirect (Table 2) pathways.

**TABLE 1:** Fit indicators for mediation models explaining the forms and functions of aggressive behavior.

		RMSEA	95% CI for RMSEA	CFI	TLI	SRMR
Model 1: reactive overt aggression	*χ*^2^_(12)_ = 19.37, *p* = 0.08	0.037	0.000; 0.066	0.97	0.95	0.025
Model 2: proactive overt aggression	*χ*^2^_(2)_ = 4.87, *p* = 0.09	0.056	0.000; 0.120	0.98	0.92	0.023
Model 3: reactive relational aggression	*χ*^2^_(10)_ = 13.77, *p* = 0.18	0.029	0.000; 0.062	0.99	0.97	0.018
Model 4: proactive relational aggression	*χ*^2^_(10)_ = 13.77, *p* = 0.14	0.032	0.000: 0.064	0.98	0.96	0.018

All baseline models were always just identified: χ2_(00)_ = 0.00, *p* = 0.000, CFI = 1.00, TLI = 1.00, SRMR = 0.000. RMSEA, root mean square error of approximation, CI, confidence interval, CFI, Comparative Fit Index, TLI, Tucker-Lewis Index, SRMR, Standardized Root Mean Square Residual.

**TABLE 2:** Indirect pathways linking early maladaptive schemas (EMS) to Social Information Processing (SIP)-related variables to the forms and functions of aggressive behavior.

	*β*	*P*
**Model 1: reactive overt aggression**		
Emotional deprivation → Hostile attribution of intent → (…)	−0.04	0.001
Subjugation → Hostile attribution of intent → (…)	−0.03	0.006
Subjugation → Evaluation of overt aggression → (…)	−0.08	< 0.001
Grandiosity → Evaluation of overt aggression → (…)	0.09	< 0.001
Insufficient self-control/discipline → Evaluation of overt aggression → (…)	0.11	< 0.001
**Model 2: proactive overt aggression**		
Subjugation → Evaluation of overt aggression → (…)	−0.06	0.001
Grandiosity → Evaluation of overt aggression → (…)	0.07	0.001
Insufficient self-control/discipline → Evaluation of overt aggression → (…)	0.09	< 0.001
**Model 3: reactive relational aggression**		
Abandonment → Hostile attribution of intent Evaluation of relational aggression → (…)	0.01	0.03
Defectiveness/Shame → Hostile attribution of intent Evaluation of relational aggression → (…)	0.02	0.03
Abandonment → Evaluation of relational aggression → (…)	−0.05	0.02
Self-sacrifice → Evaluation of relational aggression → (…)	−0.04	0.01
Grandiosity → Evaluation of relational aggression → (…)	0.04	0.02
Insufficient self-control/discipline → Evaluation of relational aggression → (…)	0.05	0.01
**Model 4: proactive relational aggression**		
Abandonment Hostile attribution of intent → Evaluation of relational aggression → (…)	0.01	0.03
Defectiveness/ Shame → Hostile attribution of intent → Evaluation of relational aggression → (…)	0.02	0.02
Abandonment → Evaluation of relational aggression → (…)	−0.05	0.02
Self-sacrifice → Evaluation of relational aggression → (…)	−0.04	0.02
Grandiosity → Evaluation of relational aggression → (…)	0.05	0.02
Insufficient self-control/discipline → Evaluation of relational aggression→ (…)	0.06	0.01

(…) denotes the dependent the outcome in each model.

#### 3.2.1 Model 1: EMSs, SIP-related variables and reactive overt aggression

The generated model concerning reactive overt aggression is depicted in Figure 1 for direct effects and Table 2 for indirect effects. The unrelenting standards, mistrust/abuse, and dependence/incompetence EMS had only direct effects on the practice of reactive overt aggression; the first two had positive effects whereas the latter had a negative effect. In other words, the more one endorses unrelating standards for ones’ own and others’ behaviors, the more one mistrusts others and the less one feels dependent on others, the more one reacts in an overtly aggressive manner. The grandiosity (i.e., believing oneself to be superior to others) and insufficient self-control/discipline (i.e., believing oneself to be unable or unwilling to manage ones’ own behavior) EMS had positive direct and indirect (through increased positive evaluation of aggression) effects on the outcome variable. The subjugation and emotional deprivation EMS had only indirect effects on reactive overt aggression, via the attribution of hostile intent. So, believing that ones’ internal experiences should be suppressed, avoided or controlled seems to be associated with hostile attribution of intent. Subjugation had also a direct and negative effect on positive evaluation of overt aggression, meaning that the less one considers the need to suppress internal experiences, the more positively they evaluated overt aggression. Finally, a negative direct effect was found between hostile attribution of intent and the practice of reactive overt aggression; alternatively, a positive direct effect was found between positive evaluation of overt aggression and the practice of reactive overt aggression.

#### 3.2.2 Model 2: EMSs, SIP-related variables and proactive overt aggression

**FIGURE 2:**
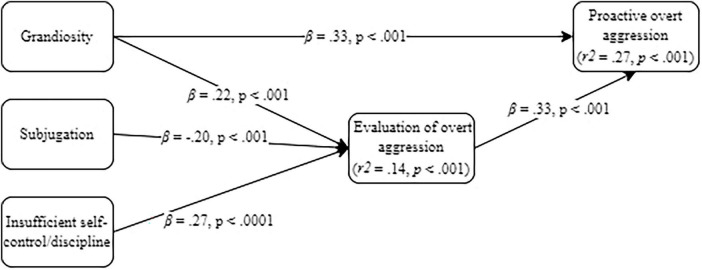
Generated mediation model on early maladaptive schemas (EMS), Social Information Processing (SIP)-related variables and proactive overt aggression.

The generated model about proactive overt aggression is depicted in Figure 2 for direct effects and Table 2 for indirect effects. The grandiosity EMS had both a direct and indirect (through a higher positive evaluation of overt aggression) effect on the outcome: the more one feels superior to others and intitled, the more they will positively evaluate and practice overt aggression. Additionally, the subjugation and insufficient self-control/discipline EMSs had only indirect effects on proactive overt aggression, though in opposite directions: believing that it is necessary to suppress ones’ internal experiences to be accepted by others impacts on less positive evaluation of overt aggression, whereas believing that one is not able or willing to control ones’ behavior in relation to social norms influenced a positive evaluation of overt aggression. Hostile attribution of intent was not a significant contribution to explaining proactive overt aggression. Alternatively, positive evaluation of overt aggression behavior had a direct and positive effect on proactively acting that way.

#### 3.2.3 Model 3: EMS, SIP-related variables and reactive relational aggression

**FIGURE 3:**
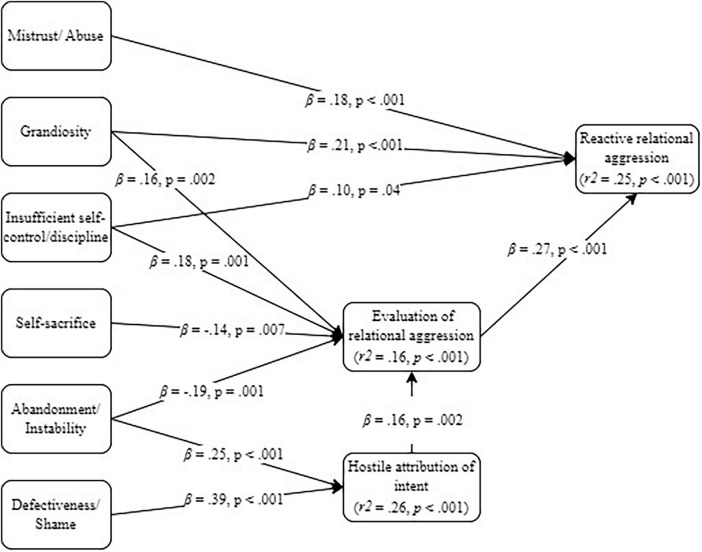
Generated mediation model on early maladaptive schemas (EMS), Social Information Processing (SIP)-related variables and reactive relational aggression.

The generated model related to reactive relational aggression is depicted in Figure 3 for direct effects and Table 2 for indirect effects. The mistrust/abuse EMS had only a direct effect on the practice of reactive relational aggression: when the individual mistrusts others as potential abusers, it is more likely that they practice that kind of aggression. The grandiosity and insufficient self-control/discipline EMSs contributed both directly and indirectly to reactive relational aggression. In other words, perceiving oneself to be superior to others and as incapable of resisting ones’ impulses or abiding by imposed limits is associated with increased practice of reactive relational aggression, both directly and through associating with a more positive evaluation of relational aggression. As for the self-sacrifice, abandonment/ instability and defectiveness/shame EMSs, they had only indirect effects on the outcome. The self-sacrifice and abandonment/instability EMSs had negative effects on positive evaluation of relational aggression, meaning that the more one believes that personal needs should be sacrificed to those of others and that others will eventually leave, abandon or reject them, the less they positively evaluate relational aggression. The abandonment/instability and defectiveness/shame EMSs also had positive effects on hostile attribution of intent, indicating that the more one believes that others will eventually leave, abandon or reject them and that there is some flaw or defect within them, the more they will attribute hostile intentions to others. Hostile attribution only impacted reactive relational aggression indirectly via increased positive evaluation of relational aggression. In turn, that response evaluation had a direct effect on practicing reactive relational aggression: when the individual positively evaluates relational aggression, it is more likely that they will practice it.

#### 3.2.4 Model 4: EMS, SIP-related variables and proactive relational aggression

**FIGURE 4:**
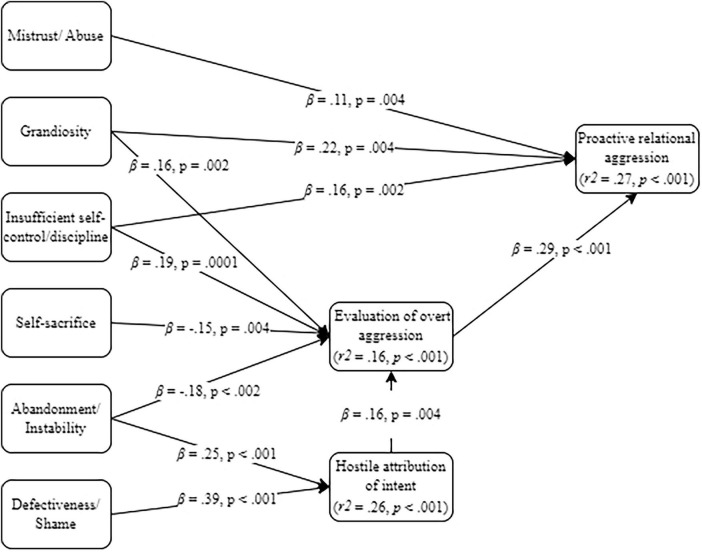
Generated mediation model on early maladaptive schemas (EMS), Social Information Processing (SIP)-related variables and proactive relational aggression.

The generated model regarding proactive relational aggression is depicted in Figure 4 for direct effects and Table 2 for indirect effects. This models’ significant direct and indirect effects were the same as those found for reactive relational aggression. So, the mistrust/abuse EMS had only a direct and positive effect on proactive relational aggression, the grandiosity and insufficient self-control/discipline EMSs had both direct and indirect (via response evaluation) positive effects in relation to proactive relational aggression, and the self-sacrifice, abandonment/ instability and defectiveness/shame EMSs had only indirect effects on that outcome, via positive evaluation of response and/or via hostile attribution of intent. Hostile attribution of intent had only an indirect effect on proactive relational aggression via being linked to increased positive evaluation of relational aggressive response. That response evaluation directly and positively impacted on practicing proactive relational aggression.

## 4 Discussion

The current work investigated the cognitive pathways underlying aggressive behavior, considered based on its forms and functions. We added to previous works (e.g., Calvete and Orue, 2012) by considering the 18 EMSs as precursors of the diverse combinations of the forms and functions of aggression. We tested four sequential mediation models wherein EMSs impacted on hostile attribution of intent (and on aggressive behavior), which in turn impacted on positive evaluation of aggression (and on aggressive behavior), which then had a direct impact on aggressive behavior (see Supplementary Figure A). Overall, our findings suggest the relevance of EMSs and of SIP-related variables to explain the enactment of aggressive behavior. Current findings also refer to specific considerations about the cognitive pathways underlying diverse ways of acting aggressively. We will discuss this general and specific findings in turn.

Overall, we confirmed the hypotheses that schemas related to the impaired limits domain were salient in impacting not only proactive and reactive overt aggression, alike previous findings (Calvete, 2008; Calvete et al., 2018b), but also relational aggression, again both reactive and proactive. So, at least on the surface, aggressive behavior seems to derive from positive perceptions of the self (Salmivalli et al., 2005), namely a perspective of oneself as deserving and unrestricted by normative social limits. Previous works have, however, referred to this grandiose and impulsive stance to be an evolutionary and adaptive response to highly threatening and devaluing developmental contexts. This means that the individuals’ presentation of themselves as if they think of themselves as worthy and entitled may be a learned way of coping (by controlling, avoiding or minimizing awareness) with negative emotional experiences (da Silva et al., 2015). Accordingly, we also found that schemas referring to the disconnection and rejection domain (i.e., mistrust/abuse, defectiveness/shame, abandonment/instability, and emotional deprivation) were relevant influencers for aggression. This aligns with previous meta-analytic findings on the association between EMS and interpersonal problems, which the authors also discuss as these schemas’ content referring specifically to interpersonal relationships (Janovsky et al., 2020). Still, EMS within the disconnection and rejection domain were currently found to impact on aggression differently for its diverse forms. Previous works had also found the relevance of this domain for explaining dating violence in adolescence (Calvete et al., 2018b) and externalizing behavior in young offenders with a clinical diagnosis (Schilder et al., 2021). These schemas may be more accurate representations of early experiences lived by individuals who report aggressive behavior (e.g., household violence, physical neglect and abuse, or emotional abuse; Stoppelbein et al., 2024) toward which they may have adopted an externalizing (instead of internalizing) way of coping. Young et al. (2006) advanced with this possibility namely within the overcompensation way of coping: aggressive individuals may use EMSs related to the impaired limits domain as a way of overcompensating for core cognitive themes related to the disconnection and rejection domains. Findings from Schilder et al. (2021) provided evidence for this assumption: adopting externalizing schema modes (e.g., losing control over angry feeling and attacking or acting impulsively to get what one needs) fully mediated the association between the disconnection/rejection domain and externalizing behavior. Proactive overt aggression seems, nonetheless, to be an exception to this cognitive pathway (see below).

Interestingly, EMSs that serve to enhance ones’ self-image (i.e., grandiosity and insufficient self-control/discipline) associated with a positive evaluation of aggressive behaviors. Instead of being aware of ones’ painful and harsh internal experiences, this justification of violence, previously associated with aggression (Calvete, 2008; Calvete and Orue, 2012), may be a continuous way of achieving cognitive consonance by justifying a learned coping violence response. Instead, EMSs that focus on being disconnected or rejected by others (i.e., defectiveness/shame or emotional deprivation) impacted on attributing a hostile intention to others. Individuals may have learnt to expect hostile interactions from others by growing up in neglectful or openly hostile environments (Stoppelbein et al., 2024) that contribute to developing a representation of oneself as unlovable and unworthy of love and care; instead, early experiences based on security, warmth and modeling of benign attributions may associate with a benign attributional style (Dodge, 2006). We propose that previous findings by Calvete and Orue (2012) may also be explained within this perspective. Specifically, attribution of intent was associated with the mistrust/abuse EMS (representing adverse childhood experiences) but not with the grandiosity EMS (representing a learned way of coping with those adverse experiences). Finally, the sequential processing of information (i.e., hostile attribution of intent impacting on aggression via a positive evaluation of aggressive behaviors) proposed by the SIP model (Crick and Dodge, 1994) was apparent only for the relational forms of aggression. Instead, for the overt forms of aggression, hostile attribution of intent either impacted directly on reactive overt aggression or was not relevant to proactive overt aggression.

A closer look at each one of the models under investigation points to specific cognitive pathways underlying relational aggression (irrespective of its function) on the one hand, and the diverse functions of overt aggression on the other. The simpler of our generated models applied to proactive overt aggression (i.e., choosing to act in an overtly aggressive way irrespective of others’ behaviors; Fiske et al., 2010); this outcome had not been considered before in relation to its underlying cognitive pathways. Proactive overt behavior was predicted directly by the grandiosity EMS and by positively evaluation of overt aggression, which in turn was explained by not being willing to control or discipline ones’ behavior nor submitting that behavior to the will of others. Hostile attribution of intent had no effect on proactive overt aggression, which aligns with previous findings (Martinelli et al., 2018). Taken together, these findings align with this function of aggression being developed through reinforcement of that behavior, which leads to continued expectations of those some rewards as consequences of acting aggressively (Dodge, 2006). If ones’ unlimited and self-serving behavior is reinforced (or at least not punished as it seems to be case for family environments associated with proactive aggression; Cima and Raine, 2009), adolescents may not only come to see it as the optimal choice but also come to see themselves as entitled to that behavior and the consequences it may grant, regardless of its cost to others.

The mediating models for relational aggression in both of its functions (reactive and proactive) were identical in what concerns EMSs and SIP-related variables that emerged as having significant effects on the outcome; the cognitive pathways leading to relational aggression had also not been detailed before. This indistinctiveness by function may be related to the fact that relational aggression, even in its reactive form, must often be delayed in relation to the perceived provocation, because relationally aggressive adolescents need to choose the right moment and the right person with whom to become allies in victimizing someone, and that often requires some time and planning. In other words, relational aggressors need to be able to process and be aware of social clues and anticipate how their actions within social networks will allow them to achieve their gains. This falls within the definition of social intelligence that seems to be particularly associated with relational (but not overt) aggression (Andreou, 2006; Björkqvist et al., 2000). It may be the case that relationally aggressive adolescents put this skill to use to cause damage to others, while non-aggressive individuals use this same skill to foster prosocial goals. Proactive and reactive relational aggression were dependent on the same EMSs, namely those within the disconnection and rejection and the impaired limits domains, as discussed above. Another similar finding for both functions of relational aggression was the sequential path from hostile attribution of intent to a positive evaluation of relational aggression, to the enactment of that behavior, sustaining the social information model premises (Crick and Dodge, 1994). In particular, the positively evaluating relational aggression in light of anticipating others’ hostile actions may reflect a learning process wherein the individual learns that being relationally aggressive serves to inflict damage without necessarily receiving the negative consequences associated with other (overt) forms of aggressive behavior (Archer and Coyne, 2005). Similarly, adolescents who are not willing to sacrifice their needs to the needs of others and believe relationships are not unstable seem to favor relationally aggressive behavior; indeed, they may expect desired outcomes from practicing these behaviors such as popularity or reciprocal relationships being maintained by excluding others (Voulgaridou and Kokkinos, 2015).

Finally, the most complex generated model concerned reactive overt aggression. A surprising finding within this generated model was the negative direct effect linking hostile attribution of intent to reactive overt aggression (i.e., the higher the attribution of hostile intent, the less practice of reactive overt aggression). Previous works have consistently associated hostile attribution of intent to both relational and overt aggression (Martinelli et al., 2018), but had not considered each form of aggression within its functions. Moreover, it seems important to look at this link based on the EMSs that supported hostile attribution of intent and then had an indirect effect on the outcome. It is interesting to note that an hostile attribution of intent was associated with the emotional deprivation and subjugation EMSs. Individuals holding these schemas are prone to either submit or alienate others, either to avoid being rejected or left out, or based on the perception that others are unavailable as sources of support, comfort or care (Rafaeli et al., 2010; Young et al., 2006). In either case, individuals holding these schemas may tend not to react in an overtly aggressive manner, even when perceiving others’ behaviors as being intentionally hostile. Other than EMSs within the disconnection and rejection and the impaired limits domains (discussed above), reactive overt aggression was directly associated with perceiving oneself to be able to function independently from others and with holding high standards for ones’ behavior. One possible way of interpreting these findings concerns the potential intermediate role of emotional arousal: interactions based on these schemas may elicit negative emotional states as one feels thwarted on ones’ independence or achievement and attributes this to others, who are consequently victims of retaliation. This would align with previous works that have associated overt aggression with emotional arousal (Cima and Raine, 2009) and emotional dysregulation (Smeijers et al., 2020), namely in relation to anger (Calvete and Orue, 2012). Still, we did not consider emotional arousal at this point, and so this inference should be taken with caution and further research into the subject is needed.

The fact that the current work did not consider emotional arousal impedes further validation of that interpretation and is one of this study’s limitations. Emotion processes have been proposed in relation to SIP early on (Lemerise and Arsenio, 2000) and empirical evidence on the association of emotional states (e.g., anger, sadness, embarrassment) and emotion dysregulation processes with hostile attribution of intent has been found (Smeijers et al., 2020). Based on the concept of schema mode, it seems plausible that EMS, attribution of intent, and emotional processes are coherently present and sustain aggressive behavior, but this remains to be addressed in future studies. Another limitation to the current work relates to its cross-sectional design that imposes caution when interpreting its findings. Though current findings mostly align with previous ones that used longitudinal designs in relation to overt aggression (e.g., Calvete and Orue, 2012), there is currently no evidence to sustain casual inferences. We also did not consider self-perpetuating cognitive and interpersonal cycles that, according to the Schema Therapy framework (Rafaeli et al., 2010; Young, 2005), may serve to maintain aggression over time. It should be relevant to conceptualize the individuals’ idiosyncratic cognitive vulnerabilities within broader ecological contexts that may inadvertently serve to sustain or reinforce those vulnerabilities (Espelage, 2014). Considering any of these aspects may have increased the variance explained by the models tested in this work, though it was still higher than that found in previous works on EMSs in relation to externalizing behaviors (e.g., Van Vlierberghe et al., 2010). Finally, we cannot ascertain that current findings would generalize to diverse samples, namely adolescents presenting with psychopathology, or adolescents presenting with both aggressor and victim experiences, though EMSs have been previously linked to externalizing behavior in young offenders (Rijo et al., 2020; Schilder et al., 2021) and to continued victimization (Calvete et al., 2018a).

Current findings have relevant clinical implications. The first implication concerns the need to carefully assess the way adolescents behave aggressively, as the applicable cognitive pathways seem different; specifically, it seems relevant to consider if the individual practices relational aggression (irrespective of its function), or overt aggression, either reactive or proactive. Then, intrapersonal therapy may ensue. Previous works have shown promising findings on managing aggressive behavior using SIP-based interventions (Dodge and Godwin, 2013) and schema therapy (Rijo et al., 2020; Van Wijk-Herbrink et al., 2017). Schema therapy in particular may benefit from targeting coping styles in relation to EMSs that may be responsible for schemas to be expressed through aggression (Janovsky et al., 2020), as well as schema modes, given that an healthy mode has been implicated in acting prosocially, even in the presence of EMS (Schilder et al., 2021). It may also be relevant to consider those interventions within a contextual and/or interpersonal approach, which has been proposed particularly to the practice of aggression (Espelage, 2014). In other words, in addition to interventions focused on the individual, interventions addressing communities where adolescents develop may also be justified, given that aggressive behavior has been associated both with teachers’ (e.g., Gini et al., 2024) and parents’ (Glatz et al., 2020) behaviors toward the adolescent. Schema Therapy assumes the relevance of changing interpersonal maladaptive cycles as was of disconfirming core cognitive themes (Rafaeli et al., 2010; Young et al., 2006), which arises as a difficult task given the stable nature of schemas and their link to neural mechanisms that sustain retrieval and schematic memory generalization of schema-congruent events (Brod et al., 2017). So, changes in aggression may come from working with adolescents and/or with those that frame the adolescents’ developmental experiences, in a valuable and joint effort to change or make flexible both EMSs and SIP-related variables that were currently found to characterize the cognitive pathways underlying the diverse forms and functions of aggression.

## Data Availability

The raw data supporting the conclusions of this article will be made available by the authors, without undue reservation.

## References

[B1] Andreou E. (2006). Social preference, perceived popularity and social intelligence: Relations to overt and relational aggression. *Schl. Psychol. Int.* 27 339–351. 10.1177/0143034306067286

[B2] Archer J. Coyne S. M. (2005). An integrated review of indirect, relational, and social aggression. *Pers. Soc. Psychol. Rev.* 9 212–230. 10.1207/s15327957pspr0903_2 16083361

[B3] Björkqvist K. Österman K. Kaukiainen A. (2000). Social intelligence − empathy = aggression? *Aggr. Violent Behav.* 5 191–200. 10.1016/S1359-1789(98)00029-9

[B4] Borges J. Vagos P. Dell’Aglio D. Rijo D. (2020). Cross-cultural validation of the young schema questionnaire for adolescents in portuguese and Brazilian samples. *Int. J. Cogn. Therapy* 13 233–250. 10.1007/s41811-020-00067-6

[B5] Brod G. Lindenberger U. Shing Y. L. (2017). Neural activation patterns during retrieval of schema-related memories: Differences and commonalities between children and adults. *Dev. Sci.* 20:e12475. 10.1111/desc.12475 29076268

[B6] Calvete E. (2008). Justification of violence and grandiosity schemas as predictors of antisocial behavior in adolescents. *J. Abnormal Child Psychol.* 36 1083–1095. 10.1007/s10802-008-9229-5 18427976

[B7] Calvete E. Orue I. (2012). Social information processing as a mediator between cognitive schemas and aggressive behavior in adolescents. *J. Abnormal Child Psychol.* 40 105–117. 10.1007/s10802-011-9546-y 21785831

[B8] Calvete E. Fernández-González L. González-Cabrera J. M. Gámez-Guadix M. (2018a). Continued bullying victimization in adolescents: Maladaptive schemas as a mediational mechanism. *J. Youth Adolesc.* 47 650–660. 10.1007/s10964-017-0677-5 28434091

[B9] Calvete E. Fernández-González L. Orue I. Little T. D. (2018b). Exposure to family violence and dating violence perpetration in adolescents: Potential cognitive and emotional mechanisms. *Psychol. Violence* 8 67–75. 10.1037/vio0000076

[B10] Cima M. Raine A. (2009). Distinct characteristics of psychopathy relate to different subtypes of aggression. *Pers. Individ. Dif.* 47 835–840. 10.1016/j.paid.2009.06.031

[B11] Crick N. R. Dodge K. A. (1994). A review and reformulation of social information-processing mechanisms in children’s social adjustment. *Psychol. Bull.* 115 74–101. 10.1037/0033-2909.115.1.74

[B12] Dodge K. A. (2006). Translational science in action: Hostile attributional style and the development of aggressive behavior problems. *Dev. Psychopathol.* 18 791–814. 17152401 10.1017/s0954579406060391 PMC2745254

[B13] Dodge K. A. Godwin J. (2013). Social-information-processing patterns mediate the impact of preventive intervention on adolescent antisocial behavior. *Psychol. Sci.* 24 456–465. 10.1177/0956797612457394 23406610 PMC3726052

[B14] Espelage D. L. (2014). Ecological theory: Preventing youth bullying. aggression, and victimization. *Theory Into Pract.* 53 257–264. 10.1080/00405841.2014.947216

[B15] Farrell A. D. Bettencourt A. F. (2020). Adolescents’ appraisal of responses to problem situations and their relation to aggression and nonviolent behavior. *Psychol. Violence* 10 312–323. 10.1037/vio0000261 33777479 PMC7989797

[B16] Fiske S. T. Gilbert D. T. Lindzey G. (2010). *Handbook of Social Psychology*, Volume 2. Hoboken, NJ: John Wiley & Sons.

[B17] Fontaine R. G. Dodge K. A. (2006). Real-time decision making and aggressive behavior in youth: A heuristic model of response evaluation and decision (RED). *Aggressive Behav.* 32 604–624. 10.1002/ab.20150 20802851 PMC2928648

[B18] Fontaine R. G. Tanha M. Yang C. Dodge K. A. Bates J. E. Pettit G. S. (2010). Does response evaluation and decision (RED) mediate the relation between hostile attributional style and antisocial behavior in adolescence? *J. Abnormal Child Psychol.* 38 615–626. 10.1007/s10802-010-9397-y 20186477 PMC3726053

[B19] Fontaine R. G. Yang C. M. Dodge K. A. Pettit G. S. Bates J. E. (2009). Development of response evaluation and decision (RED) and antisocial behavior in childhood and adolescence. *Dev. Psychol.* 45:447. 10.1037/a0014142 19271830 PMC2825107

[B20] Gini G. Angelini F. Pozzoli T. (2024). Unfair teachers, unhappy students: Longitudinal associations of perceived teacher relational unfairness with adolescent peer aggression and school satisfaction. *Front. Psychol.* 15:1321050. 10.3389/fpsyg.2024.1321050 38708022 PMC11066656

[B21] Glatz T. Lippold M. Jensen T. M. Fosco G. M. Feinberg M. E. (2020). Hostile interactions in the family: Patterns and links to youth externalizing problems. *J. Early Adolesc.* 40 56–82. 10.1177/0272431618824718 32863524 PMC7453335

[B22] Hair J. F. Black W. C. Babin B. J. Anderson R. E. (2009). *Multivariate Data Analysis*, 7th Edn. London: Pearson.

[B23] Hubbard J. A. McAuliffe M. D. Morrow M. T. Romano L. J. (2010). Reactive and proactive aggression in childhood and adolescence: Precursors, outcomes, processes, experiences, and measurement. *J. Pers.* 78 95–118. 10.1111/j.1467-6494.2009.00610.x 20433614

[B24] IBM Corp (2019). *IBM SPSS Statistics for Windows, Version 26.0.* Armonk, NJ: IBM Corp.

[B25] Janovsky T. Rock A. J. Thorsteinsson E. B. Clark G. I. Murray C. V. (2020). The relationship between early maladaptive schemas and interpersonal problems: A meta-analytic review. *Clin. Psychol. Psychotherapy* 27 408–447. 10.1002/cpp.2439 32112606

[B26] Khouwaga Yusoufzai M. Lobbestael J. (2022). “Forms and functions of aggression,” in *Clinical Forensic Psychology: Introductory Perspectives on Offending*, eds Garofalo C. Sijtsema J. J. (Berlin: Springer International Publishing), 357–375. 10.1007/978-3-030-80882-2_19

[B27] Kokkinos C. M. Karagianni K. Voulgaridou I. (2017). Relational aggression, big five and hostile attribution bias in adolescents. *J. Appl. Dev. Psychol.* 52 101–113. 10.1016/j.appdev.2017.07.007

[B28] Lemerise E. A. Arsenio W. F. (2000). An integrated model of emotion processes and cognition in social information processing. *Child Dev.* 71 107–118. 10.1111/1467-8624.00124 10836564

[B29] Little T. D. Henrich C. C. Jones S. M. Hawley P. H. (2003). Disentangling the “whys” from the “whats” of aggressive behaviour. *Int. J. Behav. Dev.* 27 122–133. 10.1080/01650250244000128

[B30] Marsee M. A. Barry C. T. Childs K. K. Frick P. J. Kimonis E. R. Muñoz L. C. (2011). Assessing the forms and functions of aggression using self-report: Factor structure and invariance of the peer conflict scale in youths. *Psychol. Assess.* 23 792–804. 10.1037/a0023369 21500922

[B31] Marsee M. A. Frick P. J. Barry C. T. Kimonis E. R. Muñoz Centifanti L. C. Aucoin K. J. (2014). Profiles of the forms and functions of self-reported aggression in three adolescent samples. *Dev. Psychopathol.* 26 705–720. 10.1017/S0954579414000339 25047293

[B32] Martinelli A. Ackermann K. Bernhard A. Freitag C. M. Schwenck C. (2018). Hostile attribution bias and aggression in children and adolescents: A systematic literature review on the influence of aggression subtype and gender. *Aggress. Violent Behav.* 39 25–32. 10.1016/j.avb.2018.01.005

[B33] Muthén L.K. Muthén B. O. (2012). *Mplus Users Guide* (7th ed.). Los Angeles, CA: Muthén & Muthén.

[B34] Nicol A. Mak A. S. Murray K. Walker I. Buckmaster D. (2020). The relationships between early maladaptive schemas and youth mental health: A systematic review. *Cogn. Therapy Res.* 44 715–751. 10.1007/s10608-020-10092-6

[B35] de Castro B. O. Veerman J. W. Koops W. Bosch J. D. Monshouwer H. J. (2002). Hostile attribution of intent and aggressive behavior: A meta-analysis. *Child Dev.* 73 916–934. 10.1111/1467-8624.00447 12038560

[B36] Polman H. Orobio, de Castro B. Koops W. van Boxtel H. W. Merk W. W. (2007). A meta-analysis of the distinction between reactive and proactive aggression in children and adolescents. *J. Abnormal Child Psychol.* 35 522–535. 10.1007/s10802-007-9109-4 17340178

[B37] Rafaeli E. Bernstein D. P. Young J. (2010). *Schema Therapy: Distinctive Features.* Milton Park: Routledge, 10.4324/9780203841709

[B38] da Silva D. R. Rijo D. Salekin R. T. (2015). The evolutionary roots of psychopathy. *Aggress. Violent Behav.* 21 85–96. 10.1016/j.avb.2015.01.006

[B39] Rijo D. Miguel R. R. Paulo M. Brazão N. (2020). The effects of the growing pro-social program on early maladaptive schemas and schema-related emotions in male young offenders: A nonrandomized trial. *Int. J. Offender Therapy Comp. Criminol.* 64 1422–1442. 10.1177/0306624X20912988 32274945

[B40] Salmivalli C. Ojanen T. Haanpää J. Peets K. (2005). “I’m ok but you’re not” and other peer-relational schemas: Explaining individual differences in children’s social goals. *Dev. Psychol.* 41 363–375. 10.1037/0012-1649.41.2.363 15769192

[B41] Santos L. Vagos P. Rijo D. (2018). Dimensionality and measurement invariance of a brief form of the young schema questionnaire for adolescents. *J. Child Fam. Stud.* 27 2100–2111. 10.1007/s10826-018-1050-3

[B42] Schilder D. L. C. van Wijk-Herbrink M. F. Groenman A. P. van den Hoofdakker B. J. (2021). The mediating role of externalising and healthy schema modes in the relationship between early maladaptive schemata and overt behaviours in adolescent boys with offending behaviours, and a comparison of their early schemata with those of typically developing boys. *Criminal Behav. Mental Health* 31 109–119. 10.1002/cbm.2192 33768636 PMC8252453

[B43] Smeijers D. Benbouriche M. Garofalo C. (2020). The association between emotion, social information processing, and aggressive behavior: A systematic review. *Eur. Psychol.* 25 81–91. 10.1027/1016-9040/a000395

[B44] Steffen P. R. Elliott C. H. Lassen M. K. Olsen J. Smith L. L. (2017). Expanding schema conceptualisation and assessment: Towards a richer understanding of adaptive and maladaptive functioning. *Australian J. Psychol.* 69 200–209. 10.1111/ajpy.12141

[B45] Stoppelbein L. McRae E. Smith S. (2024). Exploring the nexus of adverse childhood experiences and aggression in children and adolescents: A scoping review. *Trauma, Violence, & Abuse* 25, 3346–3359. 10.1177/15248380241246764 38651827

[B46] Streiner D. L. (2003). Starting at the beginning: An introduction to coefficient alpha and internal consistency. *J. Pers. Assess.* 80 99–103. 10.1207/S15327752JPA8001_18 12584072

[B100] Vagos P. Rijo D. Santos I. M. (2016). Scenes for social information processing in adolescence: Item and factor analytic procedures for psychometric appraisal. *Psychol. Assess.* 28 416–428. 10.1037/pas0000194 26214013

[B47] Vagos P. Rijo D. Santos I. M. Marsee M. A. (2014). Forms and functions of aggression in adolescents: validation of the portuguese version of the peer conflict scale. *J. Psychopathol. Behav. Assess.* 36 570–579. 10.1007/s10862-014-9421-6

[B48] Van Vlierberghe L. Braet C. Bosmans G. Rosseel Y. Bögels S. (2010). Maladaptive schemas and psychopathology in adolescence: On the utility of young’s schema theory in youth. *Cogn. Therapy Res.* 34 316–332. 10.1007/s10608-009-9283-5

[B49] Van Wijk-Herbrink M. F. Broers N. J. Roelofs J. Bernstein D. P. (2017). Schema therapy in Adolescents with disruptive behavior disorders. *Int. J. Forensic Mental Health* 16 261–279. 10.1080/14999013.2017.1352053

[B50] Vatani F. Namdarpour F. (2022). Behavioral problems in adolescents with maladaptive schemas: A qualitative study. *Biannual J. Appl. Counseling* 12 95–113. 10.22055/jac.2023.39772.1857

[B51] Voulgaridou I. Kokkinos C. M. (2015). Relational aggression in adolescents: A review of theoretical and empirical research. *Aggress. Violent Behav.* 23 87–97. 10.1016/j.avb.2015.05.006

[B52] Young J. E. (2005). *Young Schema Questionnaire S3. Cognitive Therapy Center of New York.* Washington, DC: APA.

[B53] Young J. E. Klosko J. S. Weishaar M. E. (2006). *Schema Therapy: A Practitioner’s Guide.* New York, NY: Guilford Press.

