# Supplementary material for: Cognitive pathways to the forms and functions of aggression in adolescence: the role of early maladaptive schemas and social information processing
Source: Front Psychol. 2025 Mar 13;16:1431756. doi: 10.3389/fpsyg.2025.1431756 (PMC11966403; doi:10.3389/fpsyg.2025.1431756)

| Table A: Core cognitive themes and example of items for each of the 18 Early Maladaptive Schemas, grouped within their respective domain. | | | |
| --- | --- | --- | --- |
|  |  | Cognitive core theme | Example of item |
| Disconnection and Rejection | |  |  |
|  | Emotional deprivation | The individual believes that others will not meet their emotional needs, including affection, empathy, guidance or protection. | I don’t have people to give me warmth, support and affection. |
|  | Abandonment/ Instability | The individual believes others will eventually leave, abandon or reject them. | I worry a lot about the possibility of losing people I love and need. |
|  | Mistrust/ Abuse | The individual believes others are untrustworthy and will eventually hurt of abuse them somehow. | Sooner or later, someone will betray me. |
|  | Social isolation/ Alienation | The individual believes they are different and so unable of integrating and belonging to any social group. | I don’t see life in the same way as others; I feel distant from other people. |
|  | Defectiveness/ Shame | The individual believes there is something flawed about them and feel intensely ashamed of those flaws. | No person I care about will like me if he/she gets to know my flaws and weaknesses. |
| Impaired autonomy and performance | |  |  |
|  | Failure | The individual believes that they will likely fail in whatever they strive to do or would like to achieve. | At school, most people are more capable than I am. |
|  | Dependence/ Incompetence | The individual believes they are dependent on others and are incapable of managing their daily lives on their own. | I really need the help of others to get daily things done. |
|  | Vulnerability to harm or illness | The individual believes that serious harm or illness will very likely happen to them and that protection from that harm or illness is not possible. | I feel that a disaster could strike at any moment (an earthquake, a disease, etc.). |
|  | Enmeshment/ Undeveloped self | The individual believes that their identity, needs and/or wants are dependent on a relationship with someone else. | My parents and I are very close. We know everything about each other’s life and problems. |
| Impaired limits | |  |  |
|  | Entitlement/ Grandiosity | The individual believes they are superior, special and intitled to whatever they desire. | When I want something from others, I have a lot of trouble accepting “no” as an answer. |
|  | Insufficient self-control/discipline | The individual believes they are unwilling or uncapable of controlling their behavior to abide to general society rules. | I don’t have enough willingness to force myself to do the daily boring tasks. |
| Other-directedness | |  |  |
|  | Subjugation | The individual believes their internal experiences should be suppressed so that others will not reject or mistreat the individual for having those experiences. | I my group of friends, I feel I have to do everything they want. If not, they can tease me, make fun of me, or put me aside of the group. |
|  | Self-sacrifice | The individual believes they should sacrifice their needs and wants to those of others because others’ needs are more important. | I’m the one who usually ends up taking care of the people I’m close to. |
|  | Approval/Recognition-seeking | The individual believes their intrinsic worth is dependent on others approving them. | It gives me more pleasure to achieve something if it’s something that others can notice. |
| Over-vigilance and Inhibition | |  |  |
|  | Emotional inhibition | The individual believes that expressing their internal experiences should be avoided and self-control over those experiences should be exerted. | It’s very difficult for me to show what I feel (even positive feelings like care, affection of friendship). |
|  | Unrelating standards/ Hypercriticalness | The individual believes they should attain high standards in various areas of their lives. | I must be the best in everything I do; I can’t accept second best |
|  | Negativity/ pessimism | The individual believes their lives will be filled with difficult experiences that will have catastrophic consequences. | Even when things seem to be going well, I always have the idea that it will not last long or that it was too good to be true. |
|  | Punitiveness | The individual believes that mistakes made by themselves and/or others should be severely punished | Whenever I make a mistake, I deserve to be punished. |

| Table B: Spearman correlation values of the 18 EMS in relation to SIP-related variables and to aggressive behavior | | | | | | | | |
| --- | --- | --- | --- | --- | --- | --- | --- | --- |
|  |  | Hostile attribution of intent | Evaluation/ Favoring | | Aggressive behavior | | | |
|  |  |  | Overt aggression | Relational aggression | Reactive overt aggression | Proactive overt aggression | Reactive relational aggression | Proactive relational aggression |
| Disconnection and Rejection | |  |  |  |  |  |  |  |
|  | Emotional deprivation | .33^***^ | .17^***^ | .13^**^ | .09^*^ | .16^***^ | .16^***^ | .19^***^ |
|  | Abandonment/ Instability | .32^***^ | -.08 | -.13^**^ | .08 | -.06 | .01 | .03 |
|  | Mistrust/ Abuse | .34^***^ | .16^***^ | .09 | .25^***^ | .17^***^ | .24^***^ | .26^***^ |
|  | Social isolation/ Alienation | .39^***^ | .17^***^ | .14^**^ | .10^*^ | .12^**^ | .17^***^ | .22^***^ |
|  | Defectiveness/ Shame | .42^**^ | .18^**^ | .15^**^ | .11^*^ | .15^**^ | .22^**^ | .25^**^ |
| Impaired autonomy and performance | |  |  |  |  |  |  |  |
|  | Failure | .32^***^ | .07 | .06 | .04 | .08 | .10^*^ | .16^***^ |
|  | Dependence/ Incompetence | .37^***^ | .08 | .16^***^ | .04 | .09 | .21^***^ | .20^***^ |
|  | Vulnerability to harm or illness | .24^***^ | .02 | -.04 | .12^**^ | .11^*^ | .11^*^ | .17^***^ |
|  | Enmeshment/ Undeveloped self | -.08 | -.07 | -.04 | -.02 | -.03 | -.07 | -.02 |
| Impaired limits | |  |  |  |  |  |  |  |
|  | Entitlement/ Grandiosity | .13^**^ | .34^***^ | .28^***^ | .27^***^ | .30^***^ | .37^***^ | .35^***^ |
|  | Insufficient self-control/discipline | .26^***^ | .36^***^ | .31^***^ | .29^***^ | .33^***^ | .36^***^ | .30^***^ |
| Other-directedness | |  |  |  |  |  |  |  |
|  | Subjugation | .28^***^ | .08 | .13^**^ | .05 | .09^*^ | .18^***^ | .19^***^ |
|  | Self-sacrifice | .05 | -.11^*^ | -.18^**^ | .05 | .00 | -.03 | -.02 |
|  | Approval/Recognition-seeking | .31^***^ | .24^***^ | .22^***^ | .23^***^ | .21^***^ | .33^***^ | .32^*^ |
| Over-vigilance and Inhibition | |  |  |  |  |  |  |  |
|  | Emotional inhibition | .27^***^ | .01 | .02 | .03 | .06 | .16^***^ | .10^*^ |
|  | Unrelating standards/ Hypercriticalness | .09^*^ | .07 | .05 | .14^**^ | .16^***^ | .14^**^ | .13^**^ |
|  | Negativity/ pessimism | .35^***^ | .14^***^ | .07 | .17^***^ | .12^**^ | .16^***^ | .19^***^ |
|  | Punitiveness | .17^***^ | .00 | -.02 | .06 | .08 | .03 | .08 |
| ^***^ p ≤ .001, ^**^ p < .01, ^*^ p < .05 | | | | |  |  |  |  |

Figure A: Simplified representation of the baseline mediation models


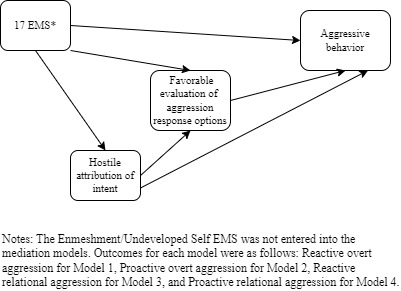

Supplement: Supplementary file 1 [file Supplementary_file_1.docx]
